# Differentiable modelling and optimization of multi-planar slicing for multi-axis additive manufacturing

**DOI:** 10.1007/s00158-025-04240-3

**Published:** 2026-01-20

**Authors:** Vibhas Mishra, Jun Wu

**Affiliations:** Sustainable Design Engineering, Faculty of Industrial Design Engineering, Delft University of Technology, Landbergstraat 15, 2628CE Delft, Zuid-Holland The Netherlands

**Keywords:** Multi-axis additive manufacturing, Multi-planar slicing, Fabrication sequence optimization, Wire arc additive manufacturing, Thermally induced distortion

## Abstract

Multi-planar deposition, enabled by multi-axis additive manufacturing, provides an opportunity to address challenging issues in wire arc additive manufacturing, such as residual stresses and distortions. This strategy involves sequentially building sub-parts, by depositing material in each sub-part with a distinct printing direction. In this paper, we present a novel continuous and differentiable formulation to model the multi-planar slicing strategy. The strategy is parameterized using a pseudo-time field, which allows the part to be segmented into sub-parts. An orientation field is used to define the distinct printing direction for each sub-part. This differentiable formulation enables gradient-based optimization of the multi-planar slicing. We apply the method to reduce distortion in wire arc additive manufacturing. The method is tested on several numerical examples with complex geometries, including holes, overhangs, and underhangs. Numerical results show that the multi-planar deposition approach reduces distortion by an order of magnitude compared with the conventional planar strategy.

## Introduction

Additive manufacturing (AM), commonly known as 3D printing, has seen rapid advancements over the past two decades. Traditionally, AM systems typically involve three translational degrees of freedom to construct 3D parts. A notable recent innovation is the integration of rotational degrees of freedom, using robotic arms or turn-tilt tables (Lettori et al. 2022). These systems, often referred to as multi-axis AM systems, offer exciting new opportunities to address challenges associated with conventional 3D printing. Central to unlocking the potential of multi-axis AM is slicing, a crucial step in transforming 3D digital models into physical parts. Slicing involves decomposing a 3D digital model into a series of layers (Livesu et al. 2017). In conventional 3D printing, the layers are planar with a fixed orientation. In contrast, multi-axis AM systems enable orientation adjustments during fabrication by rotating the build plate, the nozzle, or both.

**Fig. 1:**
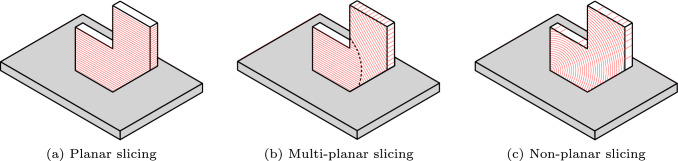
Schematic of **a** planar, **b** multi-planar, and **c** non-planar slicing for additive manufacturing

Figure 1 illustrates three slicing strategies. On the left (Fig. 1a), the layers are planar and maintain a fixed orientation. On the right (Fig. 1c), the layers are curved, and this requires continuous rotation of the build plate and/or nozzle (Dai et al. 2018; Zhang et al. 2022). This curved fabrication fully leverages the motion flexibility of multi-axis AM systems, but it introduces significant complexities in slicing and robot motion planning, such as avoiding collisions. Additionally, varying orientation and thickness along a curved layer demands continuous adjustment of process parameters (e.g., travel speed, feed rate), which presents challenges for ensuring consistent fabrication quality. In Fig. 1b, the 3D part is divided into two sub-parts, each consisting of a set of planar layers with a distinct orientation. This multi-planar slicing strategy (also referred to as multi-direction slicing) strikes a practical balance, offering enhanced flexibility over planar printing with minimal increase in complexity, and reducing the challenges associated with non-planar deposition, which may demand constant adaptation of process parameters.

In this paper, we focus on multi-planar slicing and explore its potential to address pressing challenges in robotic 3D metal printing, such as Wire-Arc Additive Manufacturing (WAAM). WAAM combines traditional welding methods with additive manufacturing principles. It is a promising technique for producing large, customized metal parts with complex geometries. However, its widespread adoption in critical industries is often hindered by residual stresses and distortions that arise during the directed energy deposition process. Previous efforts to mitigate these undesirable effects include optimizing process parameters to control thermal gradients (Rodrigues et al. 2019; Jafari et al. 2021) and post-processing, such as heat treatment to reduce residual stresses (Shen et al. 2022; Elmer et al. 2022). Despite these efforts, challenges persist in achieving a consistent quality of parts, particularly for complex geometries. In this paper, we propose addressing these challenges through a multi-planar material deposition strategy. This strategy complements the previous focus on optimizing process parameters and post-processing techniques, offering a new direction to improve the quality and precision of WAAM-produced parts.

Our objective is to provide a computational framework to optimize multi-planar slicing in order to minimize distortion caused by the fabrication process. Multi-planar slicing involves determining how a part is decomposed into sub-parts and selecting the printing direction for each sub-part. Part decomposition is a classic problem in design for additive manufacturing (Singh and Dutta 2001), and has been discussed in a few review articles (Jafari et al. 2021; Lettori et al. 2022; Oh et al. 2018). Decomposition is particularly necessary for large parts that exceed the working volume of the 3D printer (Luo et al. 2012; Yao et al. 2015; Jiang et al. 2017), as these parts must be produced in smaller segments and subsequently assembled. When combined with multi-axis printing, decomposition can reduce or even eliminate the need for support structures (Wu et al. 2017; Xu et al. 2018; Wu et al. 2019; Gao et al. 2019; Xiao and Joshi 2020). To address the relatively weaker interfaces between sub-parts, structural analysis has been used to guide the decomposition process, avoiding regions of high stress (Yao et al. 2015; Bi et al. 2023). Additionally, decomposition must consider the accessibility of the nozzle to ensure that each sub-part can be manufactured with a collision-free robotic motion (Murtezaoglu et al. 2018; Guo et al. 2025).

**Fig. 2:**
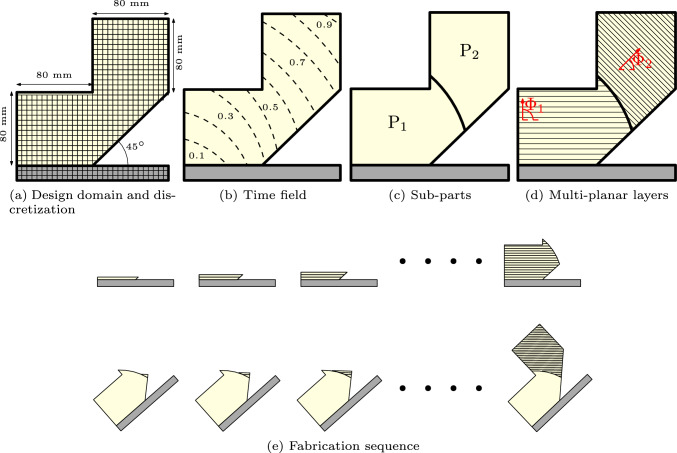
Schematic of multi-planar slicing for robotic-assisted additive manufacturing. **a** Thin-walled structure and substrate represented by finite elements. **b** Pseudo-time field indicating the fabrication sequence. **c** Sub-parts obtained by segmenting the pseudo-time field. **d** Planar layer fabrication of each sub-part with a distinct print direction. **e** Fabrication sequence of sub-parts and their respective layers. The top and bottom rows represent the fabrication of the first and second sub-parts, respectively. Note that after the completion of the first sub-part, the substrate is rotated such that the layers of the second sub-part are horizontal

Our work differentiates itself from prior research on part decomposition and multi-planar slicing in two significant ways. First, we shift the focus from manufacturability to the quality of manufacturing, and specifically aim at minimizing thermally induced distortion. Achieving this objective requires simulating the fabrication process and predicting the distortion at each iteration of the optimization process. Consequently, computational efficiency becomes critical for distortion prediction. Towards this end, we employ a simplified process model based on the inherent strain method. Originally developed for welding simulations (Ueda et al. 1975), this method substitutes computationally intensive transient thermomechanical analyses with an efficient quasi-static mechanical analysis to estimate deformation. Due to its computational efficiency, the inherent strain method has been adopted for modeling AM processes (Ma et al. 2016; Munro et al. 2019; Liang et al. 2019; Chen et al. 2019; Prabhune and Suresh 2020). It has also been successfully integrated into structural topology optimization to minimize distortion (Allaire and Jakabčin 2018; Pellens et al. 2020; Misiun et al. 2021; Miki and Yamada 2021). Unlike its typical use in structural optimization, in this work, we employ the inherent strain method to optimize the fabrication sequence.

Second, unlike prior approaches that explicitly represent segmentation boundaries and optimize them using recursive subdivision schemes (e.g., Wu et al. 2019), our method represents multi-planar slicing and the corresponding fabrication process using continuous and differentiable functions. This enables efficient gradient computation, facilitating gradient-based numerical optimization. Our framework builds on the recently developed space-time topology optimization approach and its extensions (Wang et al. 2020, 2023, 2024; Wu et al. 2024, 2025), which represents the fabrication sequence using a continuous pseudo-time field. While initially designed for curved fabrication, where segments correspond to curved layers, we adapt and extend it to multi-planar slicing. Here, each segment represents a sub-part that consists of many planar layers. Additionally, we introduce an orientation variable to encode the printing direction of each sub-part. This enriched formulation provides an optimization framework for determining the part decomposition and orientation of sub-parts. We further demonstrate the method’s extensibility by incorporating manufacturability considerations related to the shape and orientation of segmentation boundaries.

The remainder of this paper is structured as follows. Section 2 provides a detailed description of the differentiable modeling approach for multi-planar slicing. Section 3 discusses the distortion calculation and the formulation of the optimization problem. Sect. 4 compares different optimization setups and demonstrates the effectiveness of multi-planar slicing through multiple numerical examples, including consideration of manufacturability. Finally, Sect. 5 summarizes the key findings of this study.

## Multi-planar slicing

Given the design of a part, our method aims to find a decomposition of the part and the distinct print direction per sub-part. To achieve this, we introduce two sets of variables: a pseudo-time field ($\boldsymbol{\tau }$), and a set of orientation angles, $[\Phi _1, \Phi _2,..., \Phi _{\textrm{N}_\textrm{P}}]$, where $\Phi _\textrm{i}$ represents the print direction of the *i*-th sub-part, and $\textrm{N}_\textrm{p}$ is the number of sub-parts, which the user specifies.

The pseudo-time field is defined on a finite element discretization of the part ($\Omega$), as schematically shown in Fig. 2a and b. Each element takes a time value between 0.0 and 1.0, where a larger value indicates the element materializes later in the fabrication process. The purpose of the pseudo-time field is to decompose the part into sub-parts. For instance, as illustrated in Fig. 2c, the threshold of 0.5 segments the part into two separate sub-parts. The elements where the associated time values are smaller than or equal to 0.5 belong to the first sub-part ($\textrm{P}_1$), and the elements with values larger than 0.5 are in the second sub-part ($\textrm{P}_2$).

For each sub-part, the print direction uniquely defines a set of planar layers. We consider a virtual slicing plane orthogonal to the print direction. The slicing plane starts from a reference point (e.g., the bottom left of the part) and propagates along the print direction at a constant step size, generating a series of planar layers. Figure 2d illustrates the planar layers in sub-parts. The step size represents the thickness of the layer in the AM process. Figure 2e illustrates the fabrication sequence, where the top and bottom rows show the layer-by-layer deposition of the first and second sub-parts, respectively. We note that for each sub-part, the substrate can be rotated to ensure the layers remain horizontal, typically achieved using a turn-tilt table.

### Pseudo-time field

We utilize a pseudo-time field to encode the fabrication sequence. To mimic the additive process, this field must be continuous and monotonically increasing from the designated starting point (e.g., the build plate). To enforce these properties, we adopt the PDE-based regularization approach proposed by Wang et al. (2024). Instead of optimizing the pseudo-time field directly, we introduce an auxiliary field as optimization variables, from which the pseudo-time field is derived by solving a partial differential equation (PDE). Specifically, the PDE represents a fictitious thermal diffusion process: a heat source is applied to the build plate, and the temperature decreases smoothly throughout the domain. By optimizing element-wise thermal conductivities, we obtain a smooth temperature distribution, which is then interpreted as the pseudo-time field.

The PDE-based regularization works as follows. We discretize the design domain by a structured finite element mesh, and assign each element a design variable, $\xi _\textrm{e}$, representing the thermal conductivity of that element ($\textrm{e}$). The element-level thermal conductivity matrix is obtained by an interpretation function, similar to the modified SIMP commonly used in density-based topology optimization (Bendsøe and Sigmund 2004),

1 $$\begin{aligned} \{\textbf{k}_\textrm{T}\}_\textrm{e} = (\kappa _\textrm{min} + \xi _\textrm{e} (\kappa _\textrm{0}-\kappa _\textrm{min}))\{\textbf{k}_\textrm{T}\}. \end{aligned}$$

Here $\kappa _\textrm{0}$ and $\kappa _\textrm{min}$ are the maximum and minimum values of the thermal conductivity, respectively. We choose $\kappa _\textrm{0} = \textrm{1}$ and $\kappa _\textrm{min} = \textrm{1} \times {10^{-9}}$. $\{\textbf{k}_\textrm{T}\}$ is the element conductivity matrix for an element with unit conductivity. The subscript $\textrm{e}$ represents the element level matrix of element $\textrm{e}$ with the design variable $\xi _\textrm{e}$.

The heat equation describes steady-state thermal conduction in a heterogeneous medium, characterized by spatially varying thermal diffusivity and the presence of a drain term. Detailed formulations can be found in Wang et al. (2024). In its discretized form, the equation is expressed as

2 $$\begin{aligned} \textbf{K}_{\textbf{T}}\textbf{T} = \textbf{b}, \end{aligned}$$

where the global system matrix $\textbf{K}_{\textbf{T}}$ is constructed from contributions of the thermal conductivity matrix and the drain term. $\textbf{T}$ is the global nodal temperature vector, and $\textbf{b}$ is the global heat load vector. We impose Dirichlet boundary conditions on the nodes that represent the substrate domain. The temperature on these nodes is constant and takes a value of 1. The initial temperature values at other nodes are equal to 0. This results in a temperature distribution with values between 0 and 1. After solving the heat equation, we transform the temperature from nodes ($\textbf{T}$) to elements (${\mathbb {T}}$) by bilinear interpolation,

3 $$\begin{aligned} {\mathbb {T}} = \textbf{A} \textbf{T}, \end{aligned}$$

where $\textbf{A}$ is the transformation matrix. Effectively, the elemental temperature is the mean of the temperature values of its corresponding nodes.

The temperature decreases from the substrate within the domain. We reverse the order of the scalar field by defining the pseudo-time field as

4 $$\begin{aligned} \boldsymbol{\tau } = \textbf{1}- \boldsymbol{\mathbb {T}}. \end{aligned}$$

In this way, the pseudo-time near the substrate is close to zero, representing the early stage in the fabrication process.

**Fig. 3:**
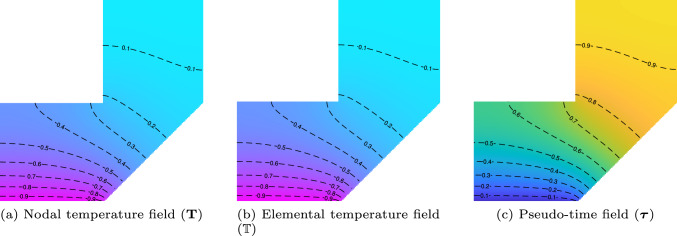
Schematic of pseudo-time field generation. **a** Nodal temperature field $\textbf{T}$, computed by solving the fictitious heat conduction problem. **b** Elemental temperature field $\mathbb {T}$, obtained via bilinear interpolation of $\textbf{T}$. **c** Pseudo-time field $\boldsymbol{\tau }$, derived through complementation $\boldsymbol{\tau } = 1 - \mathbb {T}$

To illustrate the workflow of the proposed process, we solve the heat equation on a numerical example. The nodal temperature, elemental temperature, and the pseudo-time fields, with isolines, are shown in Fig. 3. High temperature values are near the substrate and low temperatures are away from it. The pseudo-time field complements it, indicating early deposition near the substrate and later deposition away from it.

### Part decomposition

From the pseudo-time field, the part is segmented into sub-parts by prescribed timestamps. Assuming a total of $\textrm{N}_{\textrm{P}}$ sub-parts, we denote the corresponding timestamps by ${\tau }_\textrm{0}$, ${\tau }_\textrm{1}$,...${\tau }_{\textrm{N}_\textrm{P}}$. Each timestamp, ${\tau }_{\textrm{i}}$, divides the part into two regions: one that has already been fabricated up to ${\tau }_{\textrm{i}}$, and another that remains to be fabricated. For gradient-based numerical optimization, the division is realized via an inverted Heaviside function,

5 $$\begin{aligned} \textbf{H}(\boldsymbol{\tau },{\tau }_{\textrm{i}}) = 1 - \dfrac{1}{1+ \exp \left( {-{\textrm{k}_1} \left( {\boldsymbol{\tau }-{\tau _{\textrm{i}}}} \right) } \right) }. \end{aligned}$$

**Fig. 4:**
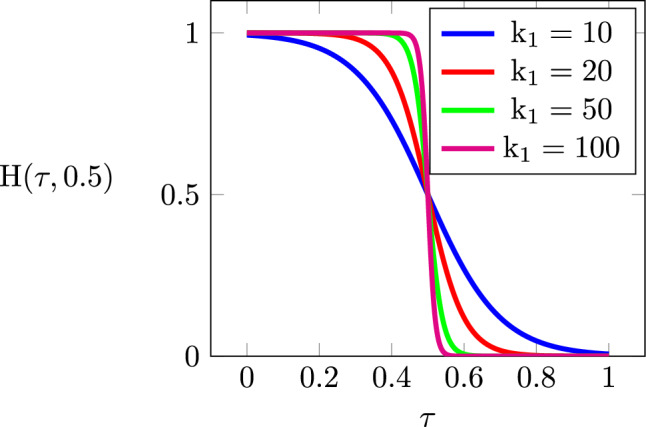
The effect of parameter $\textrm{k}_1$ on the shape of the inverted Heaviside function

This function differentially transforms a time field into a quasi-binary distribution. It maps time values smaller than the timestamp ${\tau }_{\textrm{i}}$ to values close to 1, and larger ones to values close to 0. Being continuous and differentiable, this function allows for smooth transitions, with the parameter $\textrm{k}_1$ controlling the sharpness of the Heaviside projection. Figure 4 illustrates the effect of this parameter on the function’s shape. In our study, the default value of $\textrm{k}_1$ is = 100.

The elements with pseudo-time values between two successive timestamps (${\tau }_{\textrm{i}-1}$ and ${\tau }_{\textrm{i}}$) constitute a sub-part, $\textrm{P}_\textrm{i}$. These elements are identified by the difference between the Heaviside function that takes ${\tau }_{\textrm{i}-1}$ and ${\tau }_{\textrm{i}}$ as input,

6 $$\begin{aligned} \textbf{E}_{\textrm{P}_\textrm{i}} = \textbf{H}(\boldsymbol{\tau },{\tau }_{\textrm{i}})-\textbf{H}(\boldsymbol{\tau },{\tau _{\textrm{i}-1}}). \end{aligned}$$

$\textbf{E}_{\textrm{P}_\textrm{i}}$ is again a scalar field, with values close to 1 for elements belonging to sub-part $\textrm{P}_\textrm{i}$ and 0 elsewhere.

**Fig. 5:**
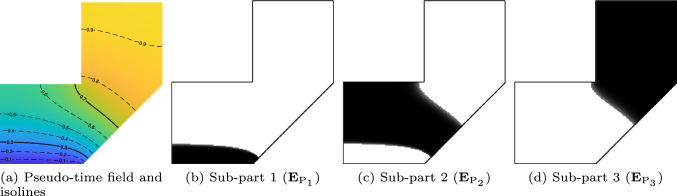
The isolines corresponding to the prescribed timestamps, indicated by the solid lines, segment the pseudo-time field (**a**), and thus the entire part, into three sub-parts (**b**, **c**, **d**)

For illustrative purposes, the previous part is divided into 3 sub-parts, by timestamps 0, 0.3, 0.7, and 1.0. In Fig. 5a, the solid isolines correspond to the timestamp values. Figure 5b-d display the three sub-parts. A smooth but narrow transition at the interface between sub-parts can be observed. They result from the continuous nature of the smoothed Heaviside function.

### Planar slicing of sub-parts

The next computational step is to slice each sub-part into planar layers along its designated print direction. This process involves two major steps. First, the domain of the entire part is sliced according to the print direction. Then, the planar layers for each sub-part are determined by restricting the slices to the sub-part’s domain.

**Fig. 6:**
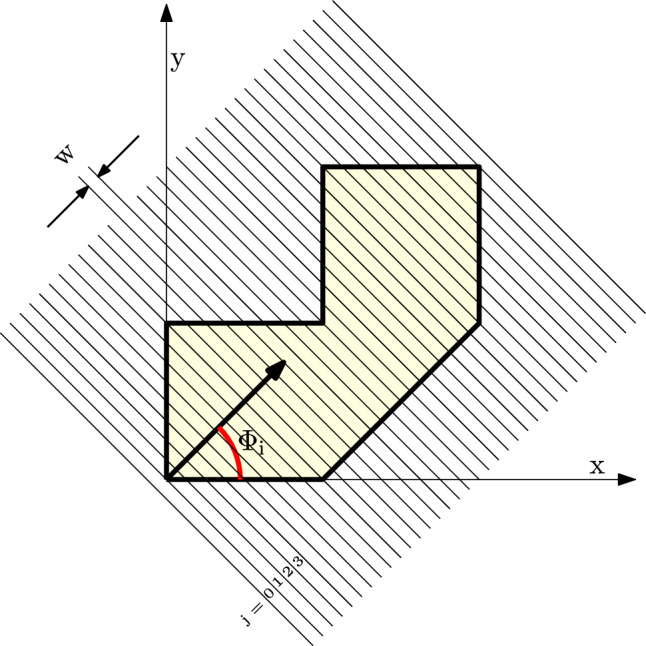
Planar slicing over the domain of the entire part, according to the print direction $\Phi _{\textrm{i}}$

As illustrated in Fig. 6, the planar layers are oriented orthogonally to the print direction, which is defined by the orientation variable $\Phi _{\textrm{i}}$, representing the angle between the print direction and the $x-$axis. Given the print direction, we define the line that passes through the origin and is orthogonal to the print direction as a reference. This reference line then sweeps through the entire domain with a constant step size, determined by a user-specified layer thickness. Depending on the print direction, the sweeping may have to proceed both along the print direction and its negative direction to cover the domain of the entire part.

The elements located between two consecutive lines form a layer. To determine this, we first compute the distance from the centroid of each element to the reference line,

7 $$\begin{aligned} {\textbf{S}_{\textrm{i}}} = \textbf{Y}\sin {\Phi _{\textrm{i}}} + \textbf{X}\cos {\Phi _{\textrm{i}}}, \quad \textrm{i} = 1, \ldots , \textrm{N}_\textrm{P}. \end{aligned}$$

Here, $\textbf{X}$ and $\textbf{Y}$ are the arrays of $\textrm{x}$ and $\textrm{y}$ coordinates of all elements, respectively. $\textbf{S}_\textrm{i}$ represents the distance from each element to the reference line corresponding to the orientation $\Phi _\textrm{i}$. We note that the distance may have positive or negative values. A negative value means that the element is below the reference line, i.e., opposite to the print direction.

From its distance to the reference line and the layer thickness, we determine in which layer an element is located. Denoting the layer index by $\textrm{j}$, elements with a distance value between $\mathrm{(j-1)}\times \textrm{w}$ and $\textrm{j}\times \textrm{w}$ fall within the $\textrm{j}-$th layer. Here, $\textrm{w}$ is the layer thickness. This is achieved through a differentiable projection function,

8 $$\begin{aligned} \textbf{E}_{\textrm{L}_{\textrm{ij}}} = 1 - \dfrac{1}{1+\textrm{exp}\bigg (\textrm{k}_2 \left( {1-\frac{4}{\textrm{w}^2}\left( {\textbf{S}_\textrm{i}-0.5\textrm{w}-\textrm{w}} \right) \left( {\textrm{j}-1} \right) } \right) ^2\bigg )}. \end{aligned}$$

**Fig. 7:**
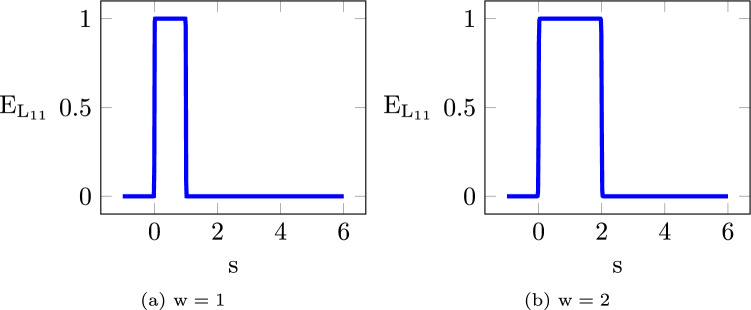
Projection function for the $\textrm{j}-$th layer ($\textrm{j}=1$ in this illustration) with a layer thickness $\textrm{w}=1$ on the left and $\textrm{w}=2$ on the right

This function projects the distance $\textrm{S}_\textrm{i}$ to 1 for values between $\mathrm{(j-1)}\times \textrm{w}$ and $\textrm{j}\times \textrm{w}$, and to $\textrm{0}$ otherwise. Like the previously introduced Heaviside function, it involves a parameter $\textrm{k}_2$, which controls the sharpness of the projection. This parameter takes a default value of $\textrm{k}_2 = 100$. Figure 7 illustrates the layer of $\textrm{j}=1$ for two different thicknesses, $\textrm{w} = 1$ on the left, and $\textrm{w} = 2$ on the right.

We note that the layer index $\textrm{j}$ is an integer between $\textrm{j}_{\textrm{min}} = \lfloor \min (\textrm{S}_\textrm{i})/\textrm{w} \rfloor$ and $\textrm{j}_{\textrm{max}} = \lceil \max (\textrm{S}_\textrm{i})/\textrm{w} \rceil$. As the distance $\textrm{S}_\textrm{i}$ may take negative values, so can the layer index $\textrm{j}$. The distance values and thus the layer index differ for each orientation variable $\Phi _\textrm{i}$. When the shape and size of the domain and the layer thickness are prescribed, the number of layers is then determined by the orientation variable. We denote the maximum number of layers by $\textrm{N}_\textrm{L}$.

After slicing the domain of the entire part, we restrict the slicing to the domain of the $\textrm{i}-$th sub-part. This is achieved by entry-wise multiplication of two fields,

9 $$\begin{aligned} \textbf{E}_{\textrm{L}_{\textrm{ij}}}^{\textrm{P}_\textrm{i}} = \textbf{E}_{\textrm{P}_\textrm{i}}\circ \textbf{E}_{\textrm{L}_{\textrm{ij}}}. \end{aligned}$$

**Fig. 8:**
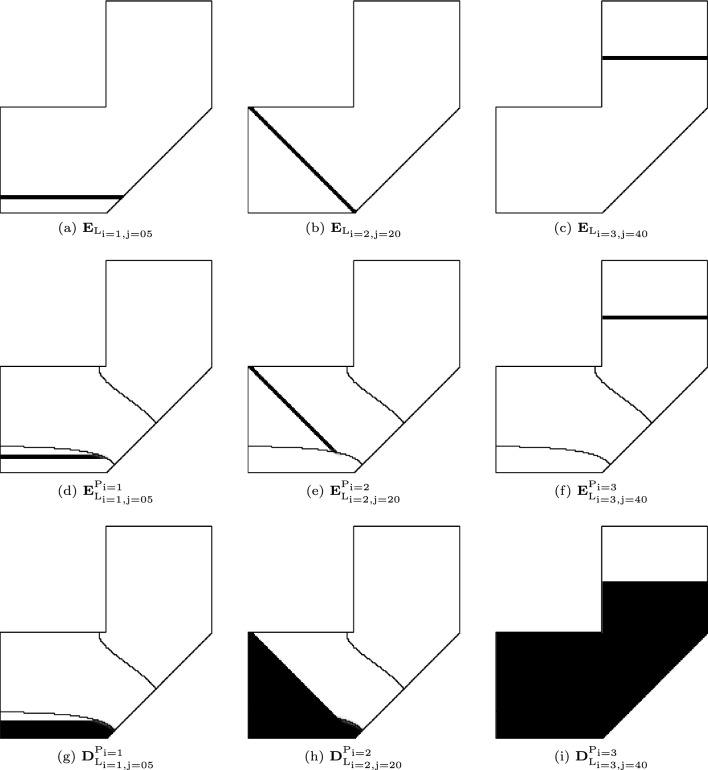
Top row: Three planar layers, each orthogonal to a distinct print direction defined over the entire domain. Middle row: The layers considering the shape and size of the sub-part. Bottom row: The intermediate structure that has been deposited up to and including the current layer

Recall that $\textbf{E}_{\textrm{P}_\textrm{i}}$ represents the $\textrm{i}-$th sub-part. $\textbf{E}_{\textrm{L}_{\textrm{ij}}}^{\textrm{P}_\textrm{i}}$ is a scalar field with values equal to 1 for elements in layer $\textrm{L}_{\textrm{ij}}$ within sub-part $\textrm{P}_\textrm{i}$, and 0 otherwise. $\textbf{E}_{\textrm{L}_{\textrm{ij}}}^{\textrm{P}_\textrm{i}}$ with $\textrm{i} \in [1, \textrm{N}_\textrm{p}]$ and $\textrm{j} \in [\textrm{j}_{\min }, \textrm{j}_{\max }]$ encodes the multi-planar slicing, i.e., both the sub-parts and successive layers in each sub-part. Figure 8 visualizes $\textbf{E}_{\textrm{L}_{\textrm{ij}}}$ and $\textbf{E}_{\textrm{L}_{\textrm{ij}}}^{\textrm{P}_\textrm{i}}$ for three layers, each in a distinct sub-part. Here, the sub-parts correspond to the decomposition in Fig. 5. The three orientation variables are ${\Phi _1} = \pi /2$, ${\Phi _2} = \pi /4$, ${\Phi _3} = \pi /2$, and the layer thickness is set to $\textrm{w} = 3~\textrm{mm}$.

## Distortion calculation and optimization problem

We employ the multi-planar slicing strategy to minimize thermally induced distortion in wire arc additive manufacturing. This section first describes the computation of the distortion, followed by the formulation of the corresponding optimization problem.

### Distortion calculation

In this work, we use the inherent strain method to simulate the thermally-induced mechanical load in the additive manufacturing process. This method assumes static mechanical loads and neglects transient thermomechanical effects due to temperature-dependent material properties. When a new layer is deposited, the inherent strain of elements belonging to this layer is activated, resulting in the deformation of previous sub-parts and layers.

The magnitude of the inherent strain is related to the thermal expansion coefficient of the material ($\alpha _{\textrm{mat}}$), and the change in temperature,

10 $$\begin{aligned} \epsilon _\textrm{ihs} = \alpha _\textrm{mat}{\mathrm{(T}_{\textrm{m}}-\textrm{T}_{0}\mathrm{)}}. \end{aligned}$$

$\textrm{T}_\textrm{m}$ and $\textrm{T}_0$ are the melting and inter-layer temperatures, respectively. Applying the inherent strain to a finite element, in Voigt notation, the strain is represented by

11 $$\begin{aligned} \boldsymbol{\epsilon }_{\textrm{ihs}} = [-\epsilon _{\textrm{ihs}}\quad -\epsilon _\textrm{ihs}\quad 0]^\textrm{T}. \end{aligned}$$

The corresponding mechanical load is calculated as

12 $$\begin{aligned} \textbf{f}_\textrm{ihs} = \textbf{B}^\textrm{T} \textbf{D}_0 \boldsymbol{\epsilon }_\textrm{ihs}. \end{aligned}$$

$\textbf{f}_\textrm{ihs}$ is the element-level load vector due to the inherent strain. $\textbf{B}$ and $\textbf{D}_0$ are the strain–displacement relationship and constitutive matrix of the material, respectively. To simulate the additive manufacturing process, we repeatedly apply the inherent strain, each time to elements associated with a new layer. Identifying the new layer by $\textbf{E}_{\textrm{L}_{\textrm{ij}}}^{\textrm{P}_\textrm{i}}$ (i.e., layer $\textrm{L}_{\textrm{ij}}$ within the sub-part $\textrm{P}_\textrm{i}$), the corresponding load vector is

13 $$\begin{aligned} \textbf{f}_{\textrm{L}_{\textrm{ij}}}^{\textrm{P}_\textrm{i}} = \bigg ({\textrm{E}_{\textrm{L}_{\textrm{ij}}}^{\textrm{P}_\textrm{i}}}\bigg )^q\textbf{f}_{\textrm{ihs}}. \end{aligned}$$

Here, *q* is a penalization coefficient. It penalizes elements with low values of ${\textrm{E}_{\textrm{L}_{\textrm{ij}}}^{\textrm{P}_\textrm{i}}}$. The assembly of element-level load vectors generates a global load vector, $\textbf{F}_{\textrm{L}_{\textrm{ij}}}^{\textrm{P}_\textrm{i}}$.

The load vector acts on the current intermediate structure that has been deposited up to and including this new layer (cf. Fig. 8 bottom row). This intermediate structure consists of previous sub-parts and previous layers within the current sub-part. Mathematically, it is identified by

14 $$\begin{aligned} {{\textbf{D}}}_{\textrm{L}_{\textrm{ij}}}^{\textrm{P}_\textrm{i}} = \textbf{H}(\boldsymbol{\tau },{\tau }_\mathrm{i-1})+\sum ^\textrm{j}_{\textrm{k}=\textrm{j}_{\min }}\textbf{E}_{\textrm{L}_{\textrm{ik}}}^{\textrm{P}_\textrm{i}}. \end{aligned}$$

On the right-hand side of the equation, the first term corresponds to the previous sub-parts, and the second term to the previous layers in the current sub-part.

The stiffness matrix of the intermediate structure is calculated according to the Solid Isotropic Material with Interpolation (SIMP) model, which is commonly used in density-based topology optimization. Specifically, the element stiffness matrix is

15 $$\begin{aligned} \mathbf {k_{\textrm{L}_{\textrm{ij}}}^{\textrm{P}_\textrm{i}}} = \left( {\textrm{E}_{\textrm{min}}} + \left( D_{\textrm{L}_{\textrm{ij}}}^{\textrm{P}_\textrm{i}}\right) ^p ({{\textrm{E}}_0} - {\textrm{E}_{\textrm{min}}}) \right) \textbf{k}_0. \end{aligned}$$

$\textrm{E}_{\textrm{min}}$ and $\textrm{E}_{0}$ are Young’s modulus values of the void and material, respectively. *p* is a penalization coefficient on the stiffness matrix. $\textbf{k}_0$ is the stiffness matrix of the material. Assembling the element-level matrices generates the corresponding global stiffness matrix, denoted as $\textbf{K}_{\textrm{L}_{\textrm{ij}}}^{\textrm{P}_\textrm{i}}$.

The (incremental) distortion of the intermediate structure, due to the mechanical load of the new layer, is obtained by solving the following state equation,

16 $$\begin{aligned} \textbf{K}_{\textrm{L}_{\textrm{ij}}}^{\textrm{P}_\textrm{i}} {\Delta } \textbf{u}_{\textrm{L}_{\textrm{ij}}}^{\textrm{P}_\textrm{i}} = \textbf{F}_{\textrm{L}_{\textrm{ij}}}^{\textrm{P}_\textrm{i}}. \end{aligned}$$

$\Delta \textbf{u}_{\textrm{L}_{\textrm{ij}}}^{\textrm{P}_\textrm{i}}$ represents the incremental distortion due to the deposition of the layer $\textrm{L}_{\textrm{ij}}$ within sub-part $\textrm{P}_\textrm{i}$. The total distortion is given as

17 $$\begin{aligned} \Delta \textbf{u} = \sum _{\textrm{i}=1\ldots \textrm{N}_\textrm{P}}\sum _{\textrm{j}=\textrm{j}_{\textrm{min}}\ldots \textrm{j}_{\textrm{max}}}\Delta \textbf{u}_{\textrm{L}_{\textrm{ij}}}^{\textrm{P}_\textrm{i}}. \end{aligned}$$

$\Delta \textbf{u}$ is the accumulation of the distortion by all layers.

**Fig. 9:**
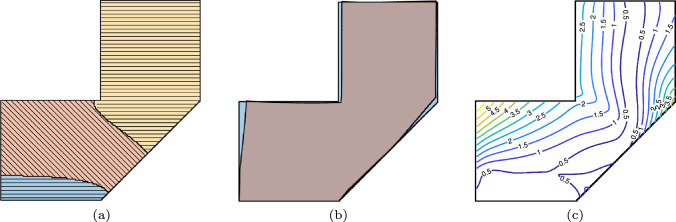
**a** The modeled multi-planar strategy using the presented differentiable mathematical formulation. **b** Predicted distorted shape (orange) superimposed on the reference shape (blue). **c** The corresponding contour plot of the magnitude of the accumulated distortion

Figure 9 illustrates the multi-planar deposition strategy and the resulting accumulated distortion from the previous numerical example. As depicted in Fig. 9a, the complete part is constructed sequentially by depositing blue-, orange-, and yellow-coloured sub-parts. Figure 9b presents the distortion according to the multi-planar sequence, where the orange and blue regions represent the distorted and reference shapes, respectively.

### Optimization problem

The proposed formulation models the multi-planar slicing and its resulting thermally-induced distortion. We introduce a gradient-based approach to optimize the multi-planar slicing in order to minimize the distortion for a given design. This strategy is parameterized by two independent sets of design variables: local conductivities ($\xi$) and orientation ($\Phi$). The optimization problem is formulated as follows.

18 $$\begin{aligned} \underset{\boldsymbol{\xi },\boldsymbol{\Phi }}{\min }&\quad \textrm{O} = \sum ^{\textrm{N}_\textrm{P}}_{\textrm{i} = 1} \sum ^{\textrm{j}_{\textrm{max}}}_{\textrm{j}=\textrm{j}_{\textrm{min}}} \bigg [\Delta \textbf{u}_{\textrm{L}_{\textrm{ij}}}^{\textrm{P}_\textrm{i}}\bigg ]^{\textrm{T}}\bigg [\Delta \textbf{u}_{\textrm{L}_{\textrm{ij}}}^{\textrm{P}_\textrm{i}}\bigg ].&\end{aligned}$$

19 $$\begin{aligned} \text {s.t.}&\quad \textbf{K}_{\textrm{L}_{\textrm{ij}}}^{\textrm{P}_\textrm{i}} \Delta \textbf{u}_{\textrm{L}_{\textrm{ij}}}^{\textrm{P}_\textrm{i}} = \textbf{F}_{\textrm{L}_{\textrm{ij}}}^{\textrm{P}_\textrm{i}}, \quad&\forall {\textrm{i} = 1 \ldots \textrm{N}_\textrm{P}},\quad \forall {\textrm{j} = 1 \ldots \textrm{N}_\textrm{L}}. \end{aligned}$$

20 $$\begin{aligned}&\textrm{g}_\textrm{i} = \dfrac{\textrm{V}_{\textrm{P}_\textrm{i}}}{\textrm{V}_0}-1\le 0, \quad&\forall {\textrm{i} = 1 \ldots \textrm{N}_\textrm{P}}. \end{aligned}$$

21 $$\begin{aligned}&0 \le {\xi _\textrm{e}} \le 1, \quad&\forall {\textrm{e} = 1 \ldots \textrm{N}_\textrm{E}}. \end{aligned}$$

22 $$\begin{aligned}&{\Phi _{\textrm{min}}} \le {\Phi _\textrm{i}} \le {\Phi _\textrm{max}}, \quad&\forall {\textrm{i} = 1 \ldots \textrm{N}_\textrm{P}}. \end{aligned}$$

Here, the objective function ($\textrm{O}$) is the aggregation of the distortion caused by every deposited layer. Equation (19) represents the state equation to determine the incremental distortion corresponding to the deposition of each new layer. Equation (20) represents the volume constraint imposed on each process interval. $\textrm{V}_{\textrm{P}_\textrm{i}}$ is the volume of the $i-$th process interval, corresponding to the field of $\textrm{E}_{\textrm{P}_\textrm{i}}$. We assume in each process interval the same amount of material is processed, i.e., $\textrm{V}_0 = \frac{1}{N_P} V_D$, where $\textrm{V}_\textrm{D}$ is the volume of the given shape. Equation (21) and Eq. (22) represent the bounds on the design variables.

To solve the optimization problem, the sensitivities of the objective function and the constraint function with respect to the design variables are required. The adjoint sensitivity analysis of the objective function is given in Appendix A.

## Results

We have implemented the proposed method in MATLAB and tested it on multiple numerical examples. This section presents the results, analyzing optimization options (Sect. 4.1), incorporating manufacturability considerations (Sect. 4.2), and demonstrating multiple examples of varying geometric complexity (Sect. 4.3). In all numerical examples, the parameters are kept consistent, taking default values listed in Table 1, unless stated otherwise.

**Table 1:** Default parameters used for the finite element modelling and optimization

Modelling and material properties		Optimization parameters	
Element size	$1~\textrm{mm}\times 1~\textrm{mm}$	*p*	3
Element Type	Plane stress Q4	*q*	3
$E_0$	$210 ~\textrm{GPa}$	$\textrm{V}_0$	$(1/\textrm{N}_\textrm{P})\textrm{V}_\textrm{D}$
$E_{\text {min}}$	$10^{-9}E_0$	$\Phi _\textrm{min}$	0
Poisson’s ratio ($\nu$)	0.3	$\Phi _\textrm{max}$	${\pi }/{2}$
Domain thickness	$1~\textrm{mm}$	$\textrm{w}$	$3~\textrm{mm}$

Thermal properties for inherent Strain calculation			
$\alpha _\textrm{mat}$	$9\times {10^{-6}}~/^\circ \textrm{C}$	$\textrm{T}_0$	$20~^{\circ } \textrm{C}$
$\textrm{T}_{\textrm{m}}$	$1500~^{\circ } \textrm{C}$		

### Optimization options

**Fig. 10:**
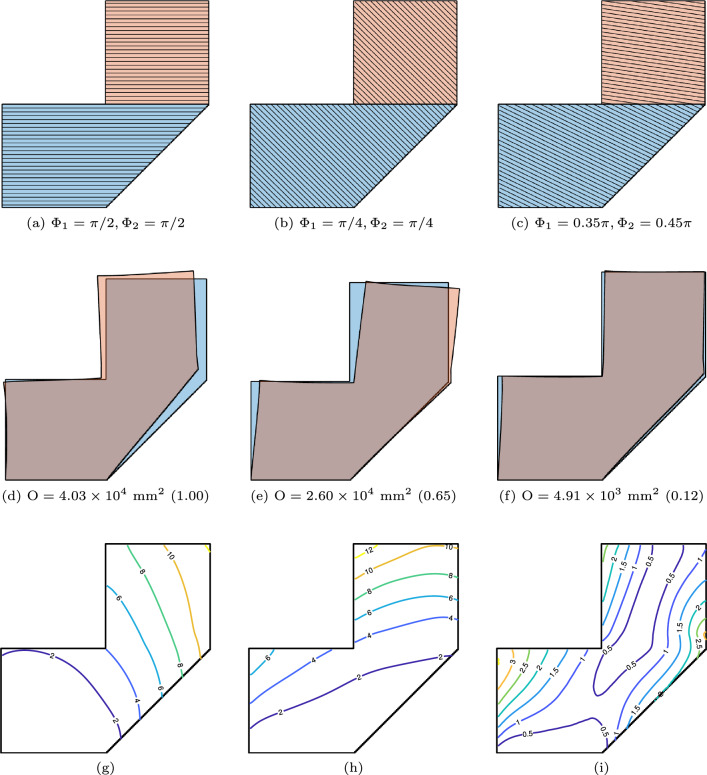
Optimization of layer orientations under a fixed segmentation. **a** and **b** illustrate two different reference orientations, while **c** presents the optimized orientations. The middle and bottom rows display the corresponding distorted shape and contour plots of accumulated distortion

The first investigation is on the effect of orientation variables when the segmentation of the parts into sub-parts remains fixed during optimization. The solution to the optimization problem yields an optimized layer orientation for each sub-part that minimizes global distortion. Figure 10 shows a prescribed segmentation of the part into two sub-parts. The sequential deposition of the blue and orange-colored sub-parts realizes the whole part. Figure 10a and b illustrate two reference orientations, while Fig. 10c depicts the optimized orientations. In the middle row, Fig. 10d–f visualize the corresponding predicted distortion. The values in parentheses indicate the normalized value of the objective function, with Fig. 10a as the reference. Compared to the horizontal layers shown on the left, the optimized layer orientation reduces distortion by $87.8\%$. The distortion from the $45^\circ$ layers is smaller than the horizontal layers but still 5.3 times larger than the optimized orientation. This comparison confirms that the orientation of the layers significantly influences the distortion objective. The bottom row, Fig. 10g–i, provides the contour plots of the accumulated distortion. Different printing strategies lead to varying patterns in the accumulated distortion, with the optimized strategy yielding a more intricate one.

**Fig. 11:**
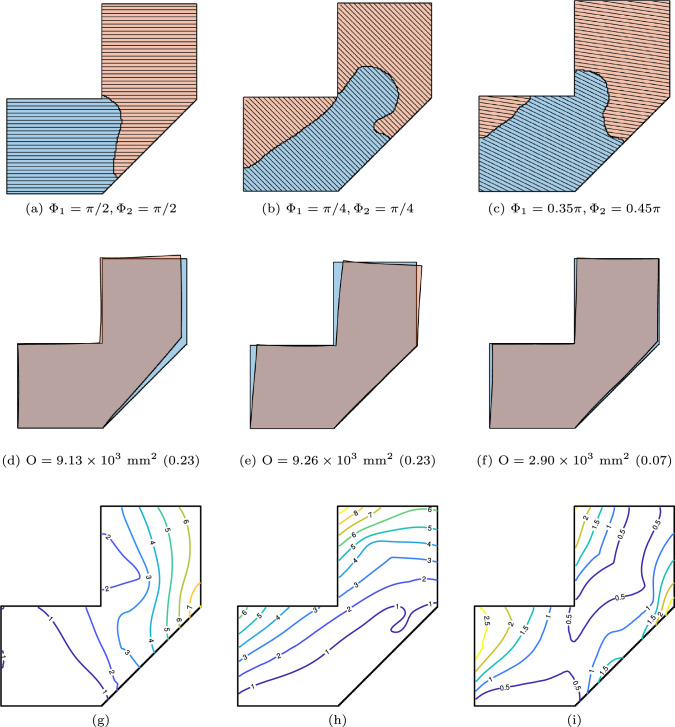
Optimization of segmentation under prescribed orientations. Top row: Optimized segmentation with the orientations taken from Fig. 10. Middle row: The corresponding distorted shapes. Bottom row: The contour plots of accumulated distortion

The next investigation is the effect of part segmentation on the distortion objective. To this end, we fix the orientation variables while allowing the local conductivities to be optimized. The fixed orientation variables are the same as those shown in Fig. 10. Figure 11 shows the optimized part segmentation and the corresponding predicted distortions. Comparing these optimized results with those in Fig. 10, we observe that optimizing the segmentation reduces the distortion by $77.3\%$, $64.4\%$, and $40.9\%$, for the three prescribed orientations, respectively. This comparison demonstrates that, even with fixed orientations, optimization of part segmentation can further reduce the distortion objective.

**Fig. 12:**
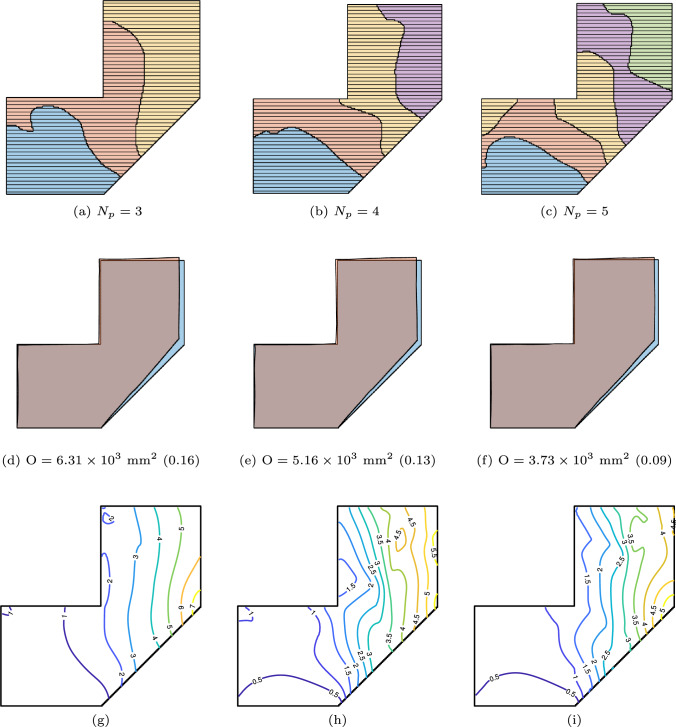
Segmentation is optimized under prescribed orientations, with the number of sub-parts increasing from left to right (3, 4, and 5, respectively). The top row presents the optimized segmentations, while the subsequent rows display the corresponding predicted distortions and their contour plots

We further analyze the effect of the number of sub-parts on distortion minimization by prescribing $\textrm{N}_\textrm{P} = 3, 4, \text {and } 5$. The orientation variables for all sub-parts are set to $\pi /2$. The optimized segmentation and predicted distortion are shown in Fig. 12. The results indicate that as the prescribed number of sub-parts increases, the distortion objective decreases. This reduction in distortion occurs as the optimization breaks the layers into shorter segments. We note that a larger number of sub-parts may not be favourable from a manufacturing perspective, as it necessitates more frequent adjustments at the transitions between sub-parts. Additionally, more complex sub-parts may increase the risk of collision issues. With more sub-parts and shorter layer lengths, the number of layers to be deposited increases, consequently raising manufacturing time. For example, the total inter-layer cooling time, required to achieve the desired temperature across all layers, is proportional to the number of layers.

**Fig. 13:**
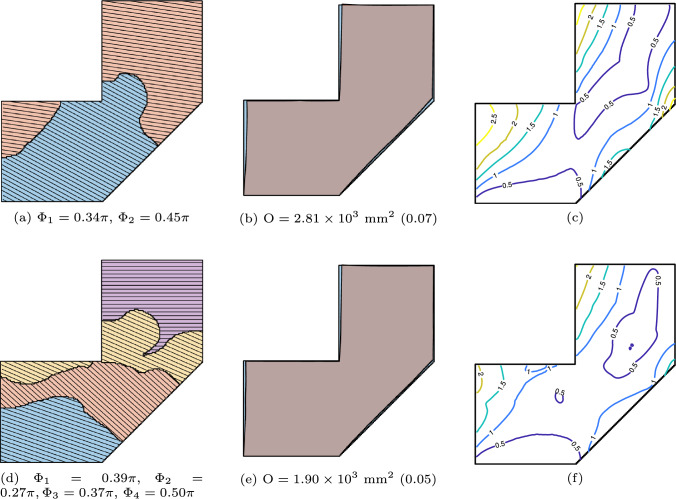
Simultaneous optimization of segmentation and orientation, with $\textrm{N}_\textrm{P} = 2$ (**a**, **b**, **c**) and 4 (**d**, **e**, **f**)

The final optimization option is the simultaneous optimization of both orientation and segmentation, providing maximum design flexibility. Figure 13 shows the results. In the upper row, simultaneous optimization is performed with $\textrm{N}_\textrm{P} = 2$. Comparing this with Fig. 10c, we observe that the simultaneously optimized result closely resembles that of the sequential optimization, while achieving a $3.1\%$ reduction in the objective function. When the part is segmented into four sub-parts, as shown in the bottom row, the objective function is further minimized.

The optimization process is computationally intensive, as each iteration requires evaluating the distortion introduced by every added layer, along with sensitivity analysis. The number of linear solver calls scales with the total number of layers, counting all sub-parts. In the numerical example of Fig. 13, with $\textrm{N}_\textrm{P}=4$ sub-parts and 120 layers in total, on a finite element grid of $160\times 160$ (52, 164 DOFs for the complete part), the wall-clock time is approximately $11~\textrm{hours}$.

### Manufacturability considerations

In the results presented in Fig. 13, the segmentation lines appear highly curved. Such curved lines lead to shorter layers at the interface, which helps to reduce the distortion objective. However, excessive curvature can create fabrication challenges, particularly regarding accessibility. To address this, we extend the framework to allow control over the shape of the segmentation lines. The key idea is to align each segmentation line with the printing direction of the subsequent sub-part. Here, the orientation of the segmentation line is derived from the gradients of the pseudo-time field, or equivalently, from the temperature gradients.

Let $\nabla \textbf{T}$ denote the gradient of the temperature field. The unit vectors $\textbf{v}$, oriented along the gradient direction, are obtained by normalizing $\nabla \textbf{T}$:

23 $$\begin{aligned} \textbf{v} = \frac{\nabla \textbf{T}}{ \left( ||\nabla \textbf{T}||_2^2 + \epsilon \right) ^{1/2}}. \end{aligned}$$

**Fig. 14:**
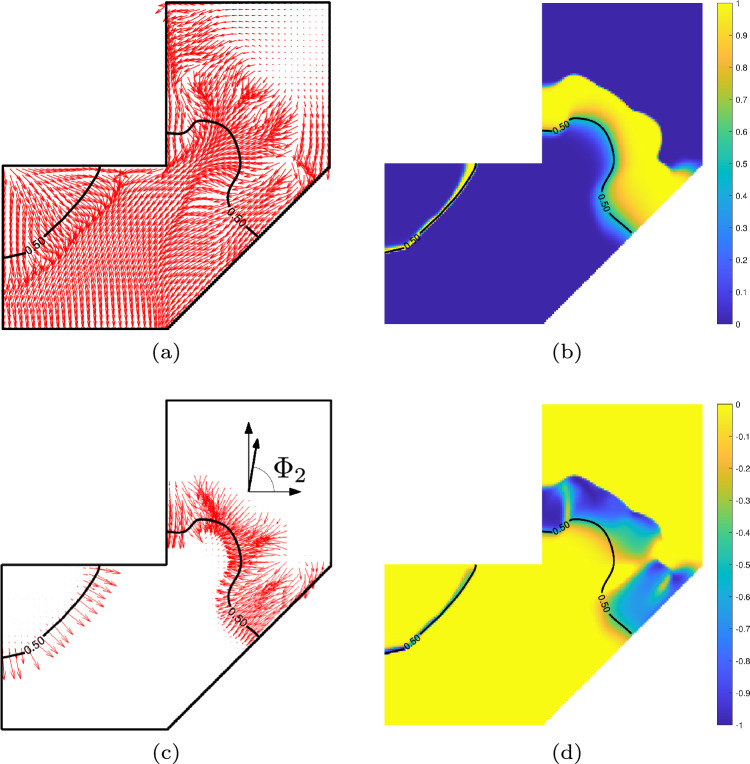
**a** Unit vectors along gradients calculated from the PDE-regularized field, $\textbf{v}$. **b** Region identified close to the segmentation line, $\mathbf {E_{B_1}}$. **c** The unit vectors of the gradients close the segmentation line. **d** Distribution of the inner product of the unit vectors of the gradients and the orientation variable, $\mathbf {C_{B_1}}$

Here, $\epsilon$ is a small positive constant introduced to prevent numerical instability when the gradient magnitude approaches zero. A default value of $1 \times 10^{-9}$ is used. Figure 14a shows the unit vectors for the case of $\textrm{N}_\textrm{P} = 2$ in Fig. 13.

Next, we extract a narrow band of elements located near the segmentation line ($B_i$) and within the subsequent sub-part. This band consists of elements whose pseudo-time values lie between $\tau _i$ and $\tau _i + \delta$, where $\delta$ is a positive constant with a default value of 0.1. In a differentiable manner, this set of elements is defined as

24 $$\begin{aligned} \textbf{E}_{B_i} = \textbf{B}(\boldsymbol{\tau }, \tau _i + \delta ) - \textbf{B}(\boldsymbol{\tau }, \tau _i), \end{aligned}$$

where $\textbf{B}(\boldsymbol{\tau }, \tau _i)$ denotes a smooth projection function. This projection maps values in $\boldsymbol{\tau }$ that are smaller than $\tau _i$ to 1 and values greater than $\tau _i$ to 0, and is given by

25 $$\begin{aligned} \textbf{B}(\boldsymbol{\tau }, \tau _i) = \frac{\tanh (k_3 \tau _i) + \tanh \bigl (k_3(\boldsymbol{\tau }-\tau _i)\bigr )}{\tanh (k_3 \tau _i) + \tanh \bigl (k_3(\textbf{1}-\tau _i)\bigr )} \end{aligned}$$

where the parameter $k_3$ controls the sharpness of the transition, with a default value of 100. Figure 14b illustrates $\textbf{E}_{B_i}$, highlighting the elements located adjacent to the segmentation line. The thickness of this band varies along the line: regions with smaller temperature gradient magnitudes produce thicker bands. Nevertheless, uniform thickness is not required for the formulation to function effectively. The corresponding unit gradient vectors within these regions are shown in Fig. 14c.

We introduce a metric to quantify the degree of (mis) alignment between the orientation of the segmentation line and the printing direction of the subsequent sub-part. Specifically, we compute the dot product between the thermal gradient vector ($\textbf{v}$) and the printing direction vector ($\boldsymbol{\eta }_{\Phi _{i+1}}$). To localize this measure to the vicinity of the segmentation line, we apply a restriction to the relevant elements, yielding

26 $$\begin{aligned} \mathbf {C_{B_i}} = \mathbf {E_{B_i}} \circ \left( \textbf{v} \cdot \boldsymbol{\eta }_{\Phi _{i+1}} \right) . \end{aligned}$$

The values of $\mathbf {C_{B_i}}$ are predominantly negative, as the thermal gradients generally point in the opposite direction to the printing direction. The distribution of $\mathbf {C_{B_i}}$ for the illustrative example is presented in Fig. 14d.

To enhance manufacturability, we introduce an additional objective that complements the primary objective of distortion minimization. Specifically, we incorporate the following terms into the objective function:

27 $$\begin{aligned} \mathrm {O_T} = \textbf{1}^\textrm{T}\mathbf {C_{B_i}} + \textbf{1}^\textrm{T}\mathbf {E_{B_i}}, \end{aligned}$$

where the first term represents the aggregated misalignment between the thermal gradients and the orientation variable. Minimizing this term promotes the alignment of the segmentation line with the printing direction. The second term aggregates the elements identified in the vicinity of the segmentation line. By minimizing this term, the total length of the segmentation line is reduced. This is important because, without such a penalty, the optimization may produce excessively long and curved segmentation lines to generate short layers, which are thermally favorable for reducing distortion. Additionally, the second term influences the magnitude of the thermal gradient, as it affects the thickness of the identified band around the segmentation line.

**Fig. 15:**
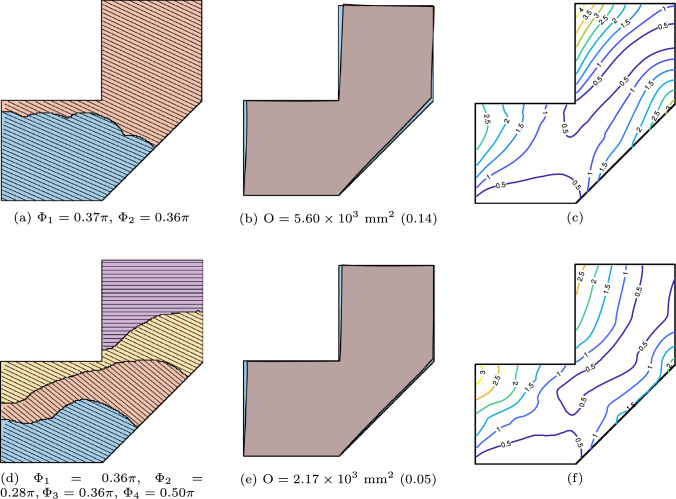
Simultaneous optimization of segmentation and orientation, with $\textrm{N}_\textrm{P} = 2$ (**a**, **b**, **c**) and 4 (**d**, **e**, **f**), considering manufacturability objective

The manufacturability objective is incorporated alongside the distortion minimization objective. The impact of this combined formulation for $N_p = 2$ and $N_p = 4$ is illustrated in Fig. 15. Compared to the baseline case shown in Fig. 13, the resulting segmentation lines exhibit reduced curvature and avoid sharp concave regions that would otherwise pose accessibility challenges.

### More examples

**Fig. 16:**
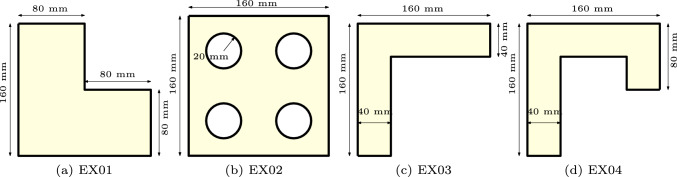
Parts of varying complexity for testing the proposed optimization framework

Further test cases are shown in Fig. 16, where the objective function has been augmented with the manufacturability considerations. We acknowledge that the chosen shapes can be fabricated without a multi-planar deposition strategy. For example, the overhang (EX03) and underhang (EX04) can be reoriented and produced using a conventional planar layer-by-layer approach. However, these shapes were selected to demonstrate the robustness of the optimization method.

**Fig. 17:**
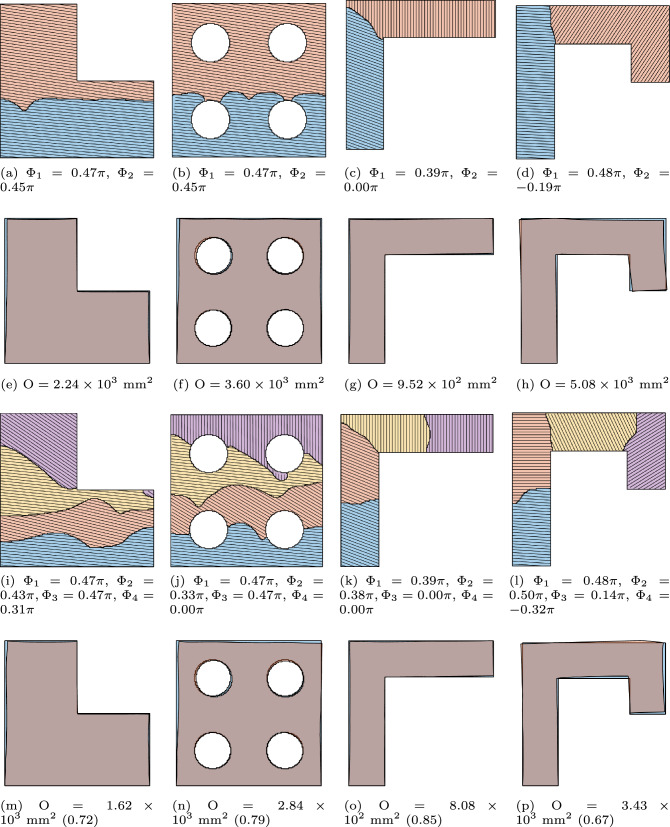
Simultaneous optimization of segmentation and orientation, with $\textrm{N}_\textrm{P} = 2$ (first and second rows) and $\textrm{N}_\textrm{P} = 4$ (third and fourth rows)

Figure 17 shows the optimized multi-planar strategies and corresponding predicted distortion, with $\textrm{N}_\textrm{P} = 2$ (first and second rows) and $\textrm{N}_\textrm{P} = 4$ (third and fourth rows). In all cases, the distorted shape (orange) closely aligns with the reference geometry (blue), demonstrating the effectiveness of the optimization approach. The objective function values in the brackets for $\textrm{N}_\textrm{P} = 4$ cases represent the normalized objective function values against their respective cases with $\textrm{N}_\textrm{P}=2$. The proposed formulation successfully addresses critical geometric features encountered during the AM process. This is most noticeable in the overhang ($\textrm{EX03}$) and underhang ($\textrm{EX04}$) cases with $\textrm{N}_\textrm{P}=2$. In both cases, a sub-part is formed by introducing a segmentation line near the top-left end of the horizontal overhanging region. The optimized layer orientation in the second sub-part makes the part more self-supporting, as numerical simulations reveal that horizontal overhangs lead to significant distortion. Furthermore, a difference in layer orientation can be observed between the overhanging regions in ($\textrm{EX03}$) and ($\textrm{EX04}$). The latter represents a more challenging scenario, for which the minimum allowable value of the orientation variable during optimization is modified from its default of 0 to $-\pi /2$. This adjustment results in an optimized layer orientation that better accommodates the underhanging feature.

**Fig. 18:**
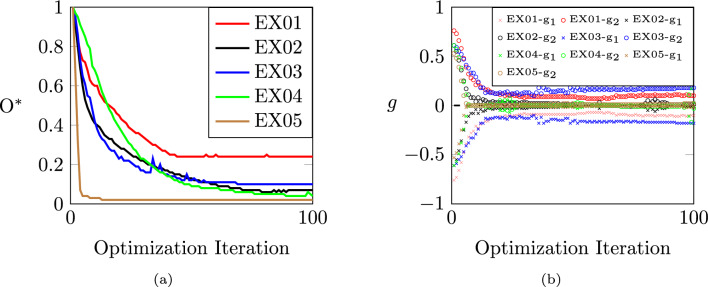
Convergence behavior of the objective function for the numerical examples with $\textrm{N}_\textrm{P} = 2$

Lastly, Fig. 18a illustrates the convergence behavior of the distortion objective for the numerical examples with $\textrm{N}_\textrm{P} = 2$. The objective function values are normalized against their initial values from the first optimization step, providing a basis for comparing the examples and a clear measure of improvement in each example. Similarly, Fig. 18b depicts the evolution of the volume constraint for each sub-part. While the constraint is initially unsatisfied, the optimization process progressively adjusts the segmentation, leading to constraint satisfaction in most cases. We observed that, in two examples, the volume constraint per sub-part was not fully satisfied at the end of the optimization. While some tuning of the optimization parameters could address this, it does not appear to be critical for practical applications. An interesting direction for future work would be to determine the volume of each sub-part through optimization.

## Conclusions

In this paper, we have presented a differentiable formulation for multi-planar slicing. This approach is inspired by recent advances in multi-axis additive manufacturing, which allows for the printing of each sub-part with a distinct printing direction. The multi-planar slicing strategy is parameterized by an orientation field and a pseudo-time field. The latter facilitates the segmentation of the part into a prescribed number of sub-parts. The formulation is applied to minimize distortion in wire arc additive manufacturing, using the inherent strain method as a simplified process model. The numerical results are encouraging. They confirm that both the printing direction and segmentation significantly influence distortion. The simultaneous optimization effectively utilizes this flexibility, resulting in a tenfold reduction in thermally induced distortion compared to the conventional planar approach.

The multi-planar strategy significantly reduces thermally induced distortion compared to conventional planar printing, while maintaining lower manufacturing complexity than curved fabrication (Wang et al. 2023). A potential manufacturing challenge arises from the emergence of curved segmentation lines. In this work, we addressed accessibility by promoting alignment between segmentation lines and the printing direction of subsequent sub-parts, as well as by minimizing their length. More research is needed to investigate further the implications of these curved segmentation lines and to develop strategies to regulate their geometry for improved manufacturability, such as considering the tool size.

## Data Availability

The relevant data, materials, and code will be made available upon publication of the article or can be provided upon request.

## Sensitivity analysis

The objective function is

A1 $$\begin{aligned} \textrm{O} = \sum _{\textrm{i} = 1 \ldots \textrm{N}_\textrm{P}} \sum _{\textrm{j}=\textrm{j}_{\textrm{min}} \ldots \textrm{j}_{\textrm{max}}} \bigg [\Delta \textbf{u}_{\textrm{L}_{\textrm{ij}}}^{\textrm{P}_\textrm{i}}\bigg ]^{\textrm{T}}\bigg [\Delta \textbf{u}_{\textrm{L}_{\textrm{ij}}}^{\textrm{P}_\textrm{i}}\bigg ]. \end{aligned}$$

Differentiating with respect to the variable $\xi _\textrm{e}$ gives

A2 $$\begin{aligned} \dfrac{\partial \textrm{O}}{\partial \xi _\textrm{e}} = \sum _{\textrm{i} = 1 \ldots \textrm{N}_\textrm{P}} \sum _{\textrm{j}=\textrm{j}_{\textrm{min}} \ldots \textrm{j}_{\textrm{max}}} 2 \bigg [\Delta \textbf{u}_{\textrm{L}_{\textrm{ij}}}^{\textrm{P}_\textrm{i}}\bigg ]^{\textrm{T}} \bigg [\dfrac{\partial \Delta \textbf{u}_{\textrm{L}_{\textrm{ij}}}^{\textrm{P}_\textrm{i}}}{\partial \xi _\textrm{e}}\bigg ]. \end{aligned}$$

The term $\bigg [\dfrac{\partial \Delta \textbf{u}_{\textrm{L}_{\textrm{ij}}}^{\textrm{P}_\textrm{i}}}{\partial \xi _\textrm{e}}\bigg ]$ is computationally expensive to calculate due to a large number of design variables ($\xi _\textrm{e}$). Therefore, an adjoint method is used here. Differentiating the state equation with respect to $\xi _\textrm{e}$ gives

A3 $$\begin{aligned} \bigg [\boldsymbol{\lambda }_{\textrm{L}_{\textrm{ij}}}^{\textrm{P}_\textrm{i}}\bigg ]^T\Bigg [\dfrac{\partial \textbf{K}_{\textrm{L}_\textrm{ij}}^{\textrm{P}_\textrm{i}}}{\partial \xi _\textrm{e}}\Delta \textbf{u}_{\textrm{L}_{\textrm{ij}}}^{\textrm{P}_\textrm{i}} + \textbf{K}_{\textrm{L}_{\textrm{ij}}}^{\textrm{P}_\textrm{i}}\dfrac{\partial \Delta \textbf{u}_{\textrm{L}_{\textrm{ij}}}^{\textrm{P}_\textrm{i}}}{\partial \xi _\textrm{e}} - \dfrac{\partial \textbf{F}_{\textrm{L}_{\textrm{ij}}}^{\textrm{P}_\textrm{i}}}{\partial \xi _\textrm{e}}\Bigg ] = \textbf{0}. \end{aligned}$$

Adding Eq. (A3) in Eq. (A2), and after simplification we get

A4 $$\begin{aligned} \dfrac{\partial \textrm{O}}{\partial \xi _\textrm{e}} = \sum _{\textrm{i}=1\ldots \textrm{N}_\textrm{P}}\sum _{\textrm{j}=1\ldots \textrm{N}_\textrm{L}} \bigg [\boldsymbol{\lambda }_{\textrm{L}_{\textrm{ij}}}^{\textrm{P}_\textrm{i}}\bigg ]^\textrm{T}\Bigg [\dfrac{\partial \textbf{K}_{\textrm{L}_{\textrm{ij}}}^{\textrm{P}_\textrm{i}}}{\partial \xi _\textrm{e}}\Delta \textbf{ u}_{\textrm{L}_{\textrm{ij}}}^{\textrm{P}_\textrm{i}} - \dfrac{\partial \textbf{F}_{\textrm{L}_{\textrm{ij}}}^{\textrm{P}_\textrm{i}}}{\partial \xi _\textrm{e}}\Bigg ]. \end{aligned}$$

The Lagrange multiplier is calculated by solving the adjoint equation,

A5 $$\begin{aligned} \textbf{K}_{\textrm{L}_{\textrm{ij}}}^{\textrm{P}_\textrm{i}}\boldsymbol{\lambda }_{\textrm{L}_{\textrm{ij}}}^{\textrm{P}_\textrm{i}} = -2\Delta \textbf{u}_{\textrm{L}_{\textrm{ij}}}^{\textrm{P}_\textrm{i}}. \end{aligned}$$

The sensitivity terms required to be evaluated are $\dfrac{\partial \textbf{K}_{\textrm{L}_{\textrm{ij}}}^{\textrm{P}_\textrm{i}}}{\partial \xi _\textrm{e}}$ and $\dfrac{\partial \textbf{F}_{\textrm{L}_{\textrm{ij}}}^{\textrm{P}_\textrm{i}}}{\partial \xi _\textrm{e}}$. Applying the chain rule, these terms can be written as

A6 $$\begin{aligned} \dfrac{\partial \textbf{K}_{\textrm{L}_{\textrm{ij}}}^{\textrm{P}_\textrm{i}}}{\partial \xi _\textrm{e}}&= \dfrac{\partial \textbf{K}_{\textrm{L}_{\textrm{ij}}}^{\textrm{P}_\textrm{i}}}{\partial {{\textbf{D}}}_{\textrm{L}_{\textrm{ij}}}^{\textrm{P}_\textrm{i}}}\dfrac{\partial {{\textbf{D}}}_{\textrm{L}_{\textrm{ij}}}^{\textrm{P}_\textrm{i}}}{\partial \boldsymbol{\tau }}\dfrac{\partial \boldsymbol{\tau }}{\partial \xi _\textrm{e}}, \end{aligned}$$

A7 $$\begin{aligned} \dfrac{\partial \textbf{F}_{\textrm{L}_{\textrm{ij}}}^{\textrm{P}_\textrm{i}}}{\partial \xi _\textrm{e}}&= \dfrac{\partial \textbf{F}_{\textrm{L}_{\textrm{ij}}}^{\textrm{P}_\textrm{i}}}{\partial {{\textbf{E}}}_{\textrm{L}_{\textrm{ij}}}^{\textrm{P}_\textrm{i}}}\dfrac{\partial {{\textbf{E}}}_{\textrm{L}_{\textrm{ij}}}^{\textrm{P}_\textrm{i}}}{\partial \boldsymbol{\tau }}\dfrac{\partial \boldsymbol{\tau }}{\partial \xi _\textrm{e}}. \end{aligned}$$

The term $\dfrac{\partial \boldsymbol{\tau }}{\partial \xi _\textrm{e}}$ is evaluated as

A8 $$\begin{aligned} \dfrac{\partial \boldsymbol{\tau }}{\partial \xi _\textrm{e}} = - \textbf{A} \dfrac{\partial \textbf{T}}{\partial \xi _\textrm{e}}. \end{aligned}$$

The adjoint method is used to evaluate $\dfrac{\partial \textbf{T}}{\partial \xi _\textrm{e}}$. The state equation is differentiated as

A9 $$\begin{aligned} \bigg [\boldsymbol{\lambda }_\textrm{T}\bigg ]^\textrm{T}\Bigg [\dfrac{\partial \textbf{K}_\textrm{T}}{\partial \xi _\textrm{e}} \textbf{T} + \textbf{K}_\textrm{T}\dfrac{\partial \textbf{T}}{\partial \xi _\textrm{e}} - \dfrac{\partial \textbf{b}}{\partial \xi _\textrm{e}}\Bigg ] = \textbf{0}. \end{aligned}$$

Since the heat load is independent of the design variable, i.e., $\dfrac{\partial \textbf{b}}{\partial \xi _\textrm{e}}=\textbf{0}$, the above equation reduces to

A10 $$\begin{aligned} \bigg [\boldsymbol{\lambda }_\textrm{T}\bigg ]^\textrm{T}\Bigg [\dfrac{\partial \textbf{K}_\textrm{T}}{\partial \xi _\textrm{e}} \textbf{T} + \textbf{K}_\textrm{T}\dfrac{\partial \textbf{T}}{\partial \xi _\textrm{e}}\Bigg ] = \textbf{0}. \end{aligned}$$

Now adding the above equation to Eq. (A8) and simplifying it leads to

A11 $$\begin{aligned} \dfrac{\partial \boldsymbol{\tau }}{\partial \xi _\textrm{e}} = - \textbf{A} \dfrac{\partial \textbf{T}}{\partial \xi _\textrm{e}} + \bigg [\boldsymbol{\lambda }_\textrm{T}\bigg ]^\textrm{T}\Bigg [\dfrac{\partial \textbf{K}_\textrm{T}}{\partial \xi _\textrm{e}} \textbf{T} + \textbf{K}_\textrm{T}\dfrac{\partial \textbf{T}}{\partial \xi _\textrm{e}}\Bigg ]. \end{aligned}$$

To evaluate the multiplier $\boldsymbol{\lambda }_\textrm{T}$, we isolate the term of $\dfrac{\partial \textbf{T}}{\partial \xi _\textrm{e}}$ and form the adjoint equation as

A12 $$\begin{aligned} \textbf{K}_\textrm{T} \boldsymbol{\lambda }_\textrm{T} = \textbf{A}^\textrm{T}. \end{aligned}$$

The sensitivity of the term $\dfrac{\partial \boldsymbol{\tau }}{\partial \xi _\textrm{e}}$ is given as

A13 $$\begin{aligned} \dfrac{\partial \boldsymbol{\tau }}{\partial \xi _\textrm{e}} = \bigg [\boldsymbol{\lambda }_\textrm{T}\bigg ]^\textrm{T}\Bigg [\dfrac{\partial \textbf{K}_\textrm{T}}{\partial \xi _\textrm{e}} \textbf{T}\Bigg ]. \end{aligned}$$

## Declarations

### Replication of Results

The authors have explained the procedure to replicate the results and intend to release the code upon publication of the article.
